# PET/CT imaging of pancreatic carcinoma targeting the “cancer integrin” αvβ6

**DOI:** 10.1007/s00259-021-05443-8

**Published:** 2021-06-09

**Authors:** Neil Gerard Quigley, Norbert Czech, Wolfgang Sendt, Johannes Notni

**Affiliations:** 1 Institute of Pathology, Technische Universität München, Trogerstr, 18, 81675 München, Germany; 2 Center of Nuclear Medicine and PET/CT Bremen, Schwachhauser Heerstraße 54, 28209 Bremen, Germany; 3 Clinic of Surgery, Hospital St. Joseph-Stift, Schwachhauser Heerstraße 54, 28209 Bremen, Germany; 4 Experimental Radiopharmacy, Clinic for Nuclear Medicine, University Hospital Essen, Hufelandstr. 55, 45147 Essen, Germany


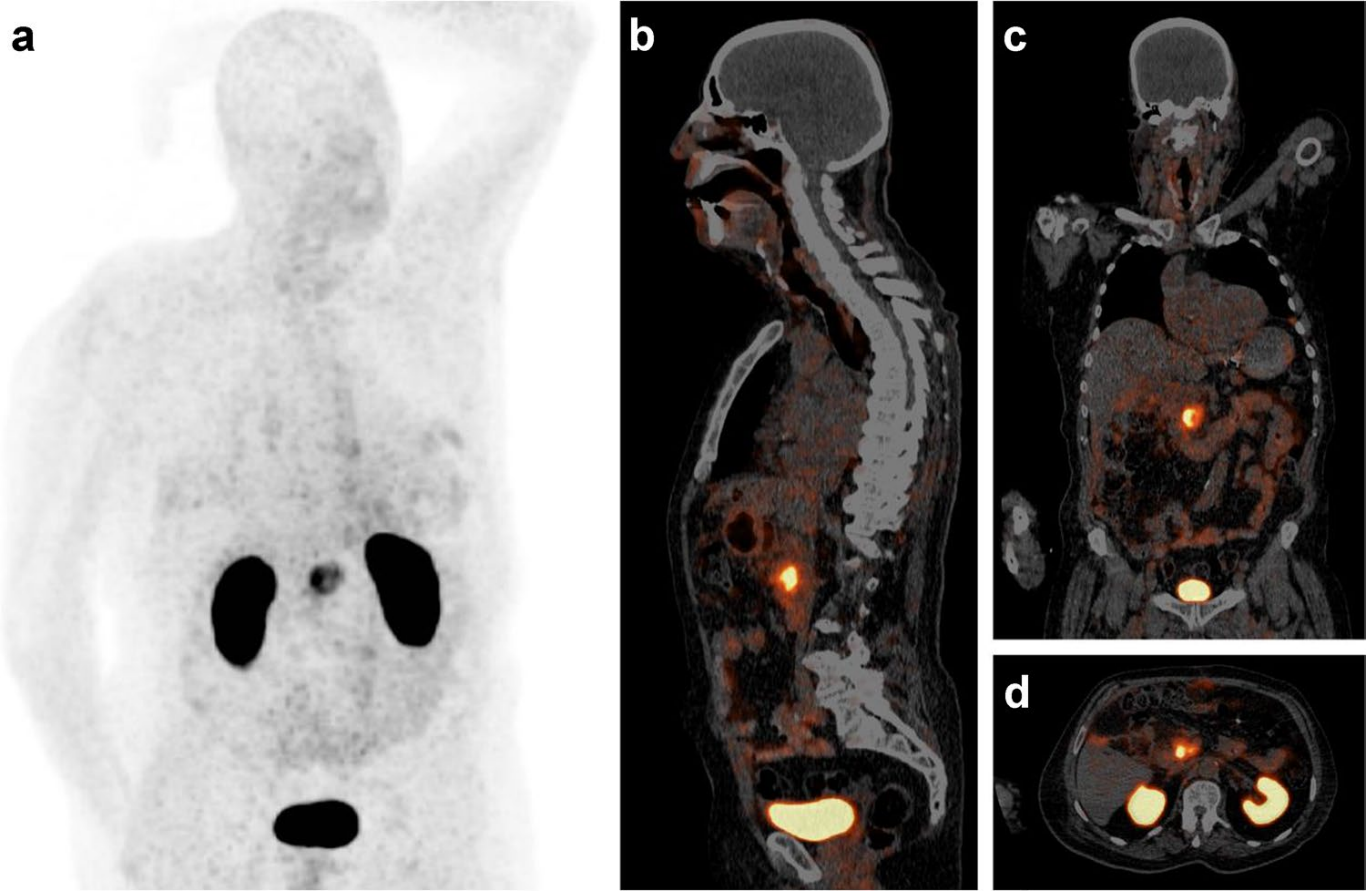


αvβ6-Integrin is exclusively expressed by epithelial cells and plays an important role for invasion and metastasis of carcinomas. We found a high expression of β6 on tumor cells in 88% of nearly 400 specimens of pancreatic ductal adenocarcinoma (PDAC) primaries and in virtually all metastases [1]. We earlier reported a series of ^68^ Ga- and ^177^Lu-labeled αvβ6-integrin-specific cyclic nonapeptides, but found that despite some showed a good tumor-to-background contrast in rodent models, tumor accumulation was ultimately too low for a successful clinical transfer [2]. We hypothesized that trimerization might result in elevated target-specific uptake and prolonged retention and thus elaborated a trimerized αvβ6-specific ^68^ Ga-peptide named ^68^ Ga-Trivehexin.

The image shows a ^68^ Ga-Trivehexin PET/CT of a male patient (82 y, 89 kg) with a histologically confirmed PDAC in the pancreatic head (87 MBq, 70 min p.i., acquisition time 25 min, 0.7 mm/s, 3 min/bed position; anterior MIP (**a**) scaled to SUV 12; PET in slices (**b**–**d**) to SUV 10). Apart from the PDAC lesion (SUV_max_ = 13.1), prominent signals are observed only in kidneys and urinary bladder due to renal excretion. No relevant uptake is seen in lungs, stomach, liver, and intestines. In light of a limited value of [^18^F]FGD-PET for early detection of PDAC [3], we anticipate that ^68^ Ga-Trivehexin will have a clinical value in this setting, besides the potential applications for fibrosis and other carcinomas (head-and-neck squamous cell, lung adenocarcinoma, colon, cervical, mammary) which have been addressed previously by αvβ6-integrin targeted PET-radiopharmaceuticals [4–6].

## Data Availability

The datasets used and/or analysed during the current study are available from the corresponding author on reasonable request.
